# Long Non-Coding RNAs as Key Modulators of Pancreatic β-Cell Mass and Function

**DOI:** 10.3389/fendo.2020.610213

**Published:** 2021-02-08

**Authors:** Livia López–Noriega, Guy A. Rutter

**Affiliations:** ^1^ Section of Cell Biology and Functional Genomics, Division of Diabetes Endocrinology and Diabetes, Department of Metabolism, Digestion and Reproduction, Imperial College London, London, United Kingdom; ^2^ Lee Kong Chian School of Medicine, Nanyang Technological University, Singapore, Singapore

**Keywords:** lncRNA, beta cell, diabetes, islet, insulin

## Abstract

Numerous studies have sought to decipher the genetic and other mechanisms contributing to β-cell loss and dysfunction in diabetes mellitus. However, we have yet to fully understand the etiology of the disease or to develop satisfactory treatments. Since the majority of diabetes susceptibility *loci* are mapped to non-coding regions within the genome, understanding the functions of non-coding RNAs in β-cell biology might provide crucial insights into the pathogenesis of type 1 (T1D) and type 2 (T2D) diabetes. During the past decade, numerous studies have indicated that long non-coding RNAs play important roles in the maintenance of β-cell mass and function. Indeed, lncRNAs have been shown to be involved in controlling β-cell proliferation during development and/or β-cell compensation in response to hyperglycaemia. LncRNAs such as *TUG-1* and *MEG3* play a role in both β-cell apoptosis and function, while others sensitize β-cells to apoptosis in response to stress signals. In addition, several long non-coding RNAs have been shown to regulate the expression of β-cell-enriched transcription factors in *cis* or in *trans*. In this review, we provide an overview of the roles of lncRNAs in maintaining β-function and mass, and discuss their relevance in the development of diabetes.

## Introduction

Diabetes Mellitus (DM) is a metabolic disease characterized by chronic hyperglycaemia, which ultimately results from pancreatic β-cell failure (1). Type 1 diabetes (T1D) involves immune cell-mediated destruction of β-cells (2), as well as more minor changes in beta cell function (3), with many patients having residual beta cell mass (4). The etiology of type 2 diabetes (T2D) remains less clear, but deficiencies in insulin secretion are a hallmark of the disease (5). T2D usually ensues when β-cells are unable to overcome insulin resistance and so maintain physiological glucose levels (6). However, a subset of patients displays defective insulin secretion despite near-normal insulin sensitivity (7) and monogenic forms of the diabetes (maturity onset diabetes of the young; MODY) involve mutations in genes which impact β-cell function (8) with unaltered insulin action.

A change in β-cell “identity” (9, 10) is a likely possibility in all forms of diabetes, though more importantly in T2D than in T1D. Numerous studies have tried to decipher the genetic and molecular mechanisms contributing to β-cell identity loss (11). Fully functional β-cells require the expression of a subset of specific transcription factors (e.g., *Pdx1, Mafa*, and *Pax6*), as well as genes involved in glucose sensing and metabolism (e.g., *Glut2/Slc2a2*, Glucokinase, *Gck)* and insulin production (insulin, *Ins*) (10, 11). Another important feature of these cells is the repression of the so-called “disallowed” genes (e.g., *Ldha*, *Hsd11b1*). Expression of this group of genes, whose transcripts are present at very low levels in β-cells compared to other tissues, may otherwise interfere with insulin secretion (12, 13).

Altered intercellular coupling between β-cells is implicated in both T2D (14, 15) and T1D (16). In both diseases, the drivers for the above changes in β-cells, and their interconnections, are likely to be both genetic and environmental, but in neither case are they fully characterized.

## Potential Roles for Non-Coding RNAs: Overview

**Figure 1:**
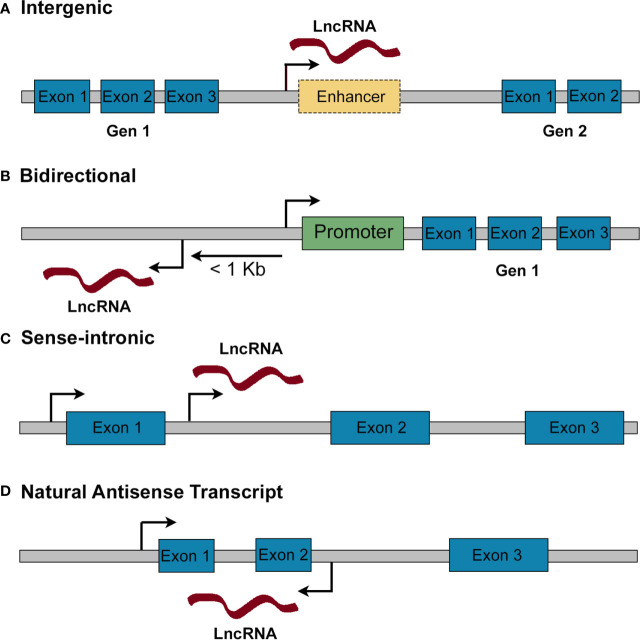
Classification of lncRNAs according to their genomic position. **(A)** Intergenic lncRNAs are found in gene deserts between two protein coding genes. They may be subclassified into long intergenic RNAs and enhancer RNAs (when they are transcribed from an enhancer). **(B)** Bidirectional lncRNAs are mapped within 1 kb from the promoter of a protein coding gene and are transcribed in the opposite direction. **(C)** Sense-intronic lncRNAs are transcribed from an intron of a protein coding gene in the same direction. **(D)** Natural antisense transcripts are transcribed from the opposite strand of a protein coding gene, overlapping partially or totally with its exons/introns.

Genome wide-association studies (GWAS) have revealed that the majority of diabetes (both T1D and T2D) susceptibility *loci* map outside protein-coding regions, consistent with a role for regulatory regions [i.e., enhancers or promoters which control the expression of protein-coding genes (17)] but also suggesting an important role for non-coding RNAs (ncRNAs) in maintaining a functional β-cell mass (18–23). NcRNAs are a heterogenous group characterized by the lack of protein coding potential, and which include both linear and circular transcripts. They can be classified according to their size into short non-coding RNAs, which are <200 nucleotides (nts) (e.g., miRNAs 19-21 nts, snRNA ~150 nts, snoRNAs 60–140 nts, piRNA 26–31 nts) and long non-coding RNAs (>200 ntds) (24, 25). Long non-coding RNAs (lncRNAs) are further classified, according to their position relative to protein coding genes, into: i) intergenic, located between two protein coding genes, usually in enhancer regions; ii) bidirectional, transcribed in the opposite direction of a protein coding gene and within 1kb of its promoter region; iii) natural antisense transcripts, RNAs overlapping partially or totally with a protein coding gene and transcribed from its opposite strand; iv) sense intronic RNAs, transcribed from intronic regions of protein coding genes in the same direction (26–28) (**Figure 1**).

LncRNAs share common features with mRNAs as they are usually transcribed by RNA polymerase II, display chromatin marks typical of active transcription and undergo post-transcriptional modifications such as 5’capping, splicing, and polyadenylation (29). However, lncRNAs are normally expressed at lower levels, contain fewer exons and are less evolutionarily conserved between species than protein-coding genes. Moreover, lncRNAs are expressed in a highly cell-type specific manner, making them well placed to be involved in cell lineage specification (29, 30). Moran et al. identified more than 1,100 lncRNAs expressed in human islets with 55% of intergenic lncRNAs and 40% of antisense transcripts being islet-specific (31). These authors also found that the expression of several of the islet-specific lncRNAs was modulated during pancreatic development, indicating that they are involved in the differentiation process of pancreatic endocrine cells. Moreover, several studies have reported differential expression of lncRNAs in islets from T1D (32) and T2D mouse models (33–35) as well as in patients with T2D (31, 36). In addition, differentially expressed lncRNAs have also been found in peripheral blood mononuclear cells from T1D patients, being proposed as biomarkers for early diagnosis (37). Therefore, it is tempting to speculate that lncRNAs are crucial players in the development of both T1D and T2D and could be used as novel biomarkers or targets for future therapies. In this review, we provide an overview of the roles of lncRNAs in maintaining β-function and mass and discuss their relevance in DM development.

## Functions of lncRNAs

**Figure 2:**
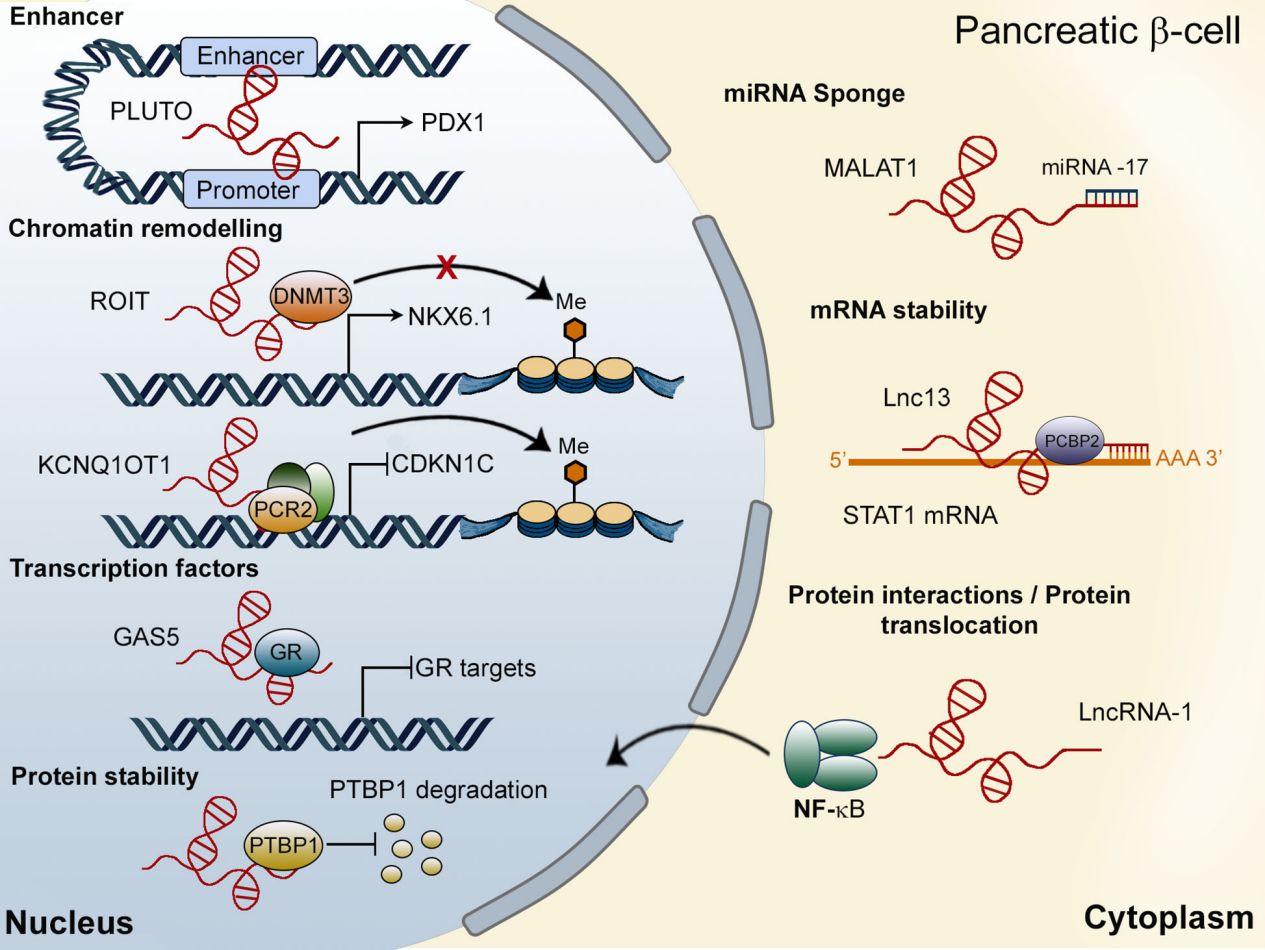
Mechanisms of action of lncRNAs in pancreatic β-cells. In the nucleus, lncRNAs, such as PLUTO, regulate the transcription of target genes (PDX1) by modulating enhancer/promoter interactions. Other LncRNAs, including ROIT and KCNQ1OT1, interact with chromatin remodeling proteins. The lncRNA GAS5 has been shown to act as a decoy for the glucocorticoid receptor (GR), inhibiting the expression of GR target genes. Finally, MALAT1 has been found to be located in the nucleus (regulating PTBP1 protein stability) as well as in the cytoplasm of β-cells, where it interacts with miRNA-17. Cytosolic lncRNAs can also modulate mRNA stability or affect protein-protein interactions or their subcellular localization.

LncRNAs can be located in the nucleus or the cytoplasm and regulate the expression of protein-coding genes both transcriptionally and post-transcriptionally (**Figure 2**) (38). In the nucleus, long non-coding RNAs have been found to modulate transcription through their interaction with chromatin remodeling proteins, recruiting, or sequestering transcription factors to their target sites and interacting with enhancer regions. Moreover, several nuclear lncRNAs can also affect alternative splicing of protein-coding genes or regulate protein stability in the nucleus (39, 40). In the cytoplasm, lncRNAs may regulate translation, control mRNA stability, act as sponges for miRNAs or affect protein-protein interactions (41). Although, classically, lncRNAs were thought to be enriched in the nucleus, a growing body of evidence suggests that they may be just as abundant in the cytoplasm (42–44). Indeed, and in contrast to previous publications (42, 45), Heesch et al. (46). reported an enrichment of lncRNAs in the cytoplasm and ribosomal fractions compared to the nucleus. Interestingly, several of the well-established nuclear lncRNAs have also been found to play important roles in the cytoplasm, suggesting that many lncRNAs are, in fact, bi-compartmental (40, 47–49). A good example is provided by *HOTAIR*, first described as a lncRNA involved in the transcriptional silencing of the HOXD cluster by the modulation of histone methylation patterns (50, 51). Nevertheless, a later study (47) reported the presence of *HOTAIR* in the cytoplasm, where it binds to two E3 ubiquitin ligases (Mex3b and Dzip3), acting as a scaffold to promote protein ubiquitination. Similarly, *MALAT1* was first described as a nuclear lncRNA involved in alternative splicing (52). However, numerous studies in different cell types have reported a function of *MALAT1* in the cytoplasm, where it acts as a sponge for miRNAs (49). In β-cells, *MALAT1* has been reported to modulate active histone marks of the *Pdx1* promoter in MIN6 cells and mouse islets (53) and to promote the stability of polypyrimidine tract binding protein 1 (Ptbp1) protein in the nucleus. Additionally, *MALAT1* is implicated in the deleterious effects of cigarette smoke on the β-cell, acting in the cytoplasm through the inhibition of *miRNA*-17 (54) (**Figure 2**). In summary, although subcellular localization has been regarded as a main determinant of the functions of a given lncRNA, a substantial body of recent evidence suggests that several lncRNAs have multiple actions in different cell compartments even within the same cell lineage.

## LncRNAs in β-Cell Proliferation

Regeneration and replacement of β-cells have been proposed as promising strategies for the treatment of insulin-requiring and insulin-dependent diabetes (55, 56). An increase in β-cell proliferation is well documented in animal models of T1D (57, 58) and T2D (59) as well as during pregnancy in rodents (60), while it remains controversial whether β-cell proliferation is significant in the human pancreas (61). However, several studies have provided evidence of β-cell proliferation in patients with recent onset T1D (62, 63). In addition, β-cell mass increases in humans during obesity and pregnancy, although immunohistochemical studies suggest that neogenesis rather than proliferation may be the main source of new β-cells in humans (64–66). The lack of unequivocal and sensitive markers to measure proliferation may complicate the task of assessing the real rate of human β-cell proliferation in this context (67). As opposed to earlier studies, Hanley et al. (68) observed increased proliferation rates using cell nuclear antigen (PCNA) staining in obese non-diabetic individuals when compared with lean non-diabetic controls. The authors also found decreased β-cell proliferation in islets from T2D donors, while a more recent study (69) indicated increased numbers of PCNA-positive β-cells in specimens from individuals with T2D when compared to nondiabetic subjects. Overall, it can be concluded that mitogenic signalling pathways are difficult to activate in human β-cells, but we still do not fully understand the mechanisms involved in β-cell proliferation and β-cell mass compensation in humans (70). Of note, recent findings point to inhibition of the protein kinase DYRK1A as a possible target for the activation of β-cell growth (71), though no evidence exists at present of a role for lncRNAs in the control of this enzyme.

In order to understand the molecular mechanisms driving β-cell mass compensation during insulin resistance or in pre-diabetes, Sisino et al., combined a NCodelncRNA Microarray with analyses from published RNA-seq data to identify lncRNAs that were differentially expressed in islets from female mice at day 14.5 of gestation, corresponding with the peak in β-cell mass expansion. The authors identified six lncRNAs that were differentially expressed in pancreatic islets during gestation. Interestingly, one of them, *Gm16308*, was strongly induced by prolactin treatment and was required for β-cell proliferation induced by the hormone. Moreover, overexpression of *Gm16308* alone was sufficient to induce β-proliferation (72), suggesting that it may be a good target to increase β-cell mass, although the impact of Gm16308 in human β-cell proliferation needs to be validated. The lncRNA DANCR (Differentiation Antagonizing Non-Protein Coding RNA) may also be involved in the compensation of β-cell mass during gestation. DANCR has been shown to act as a sponge for miR-33a-5p, which is upregulated in blood samples from patients with gestational diabetes mellitus (GDM). Overexpressing miR-33a-5p in INS-1 cells inhibited β-cell proliferation, insulin content and secretion at both low and high glucose. Remarkably, forced expression of DANCR rescued miR-33a-5p-mediated inhibition effects on cell proliferation and insulin content.

**Figure 3:**
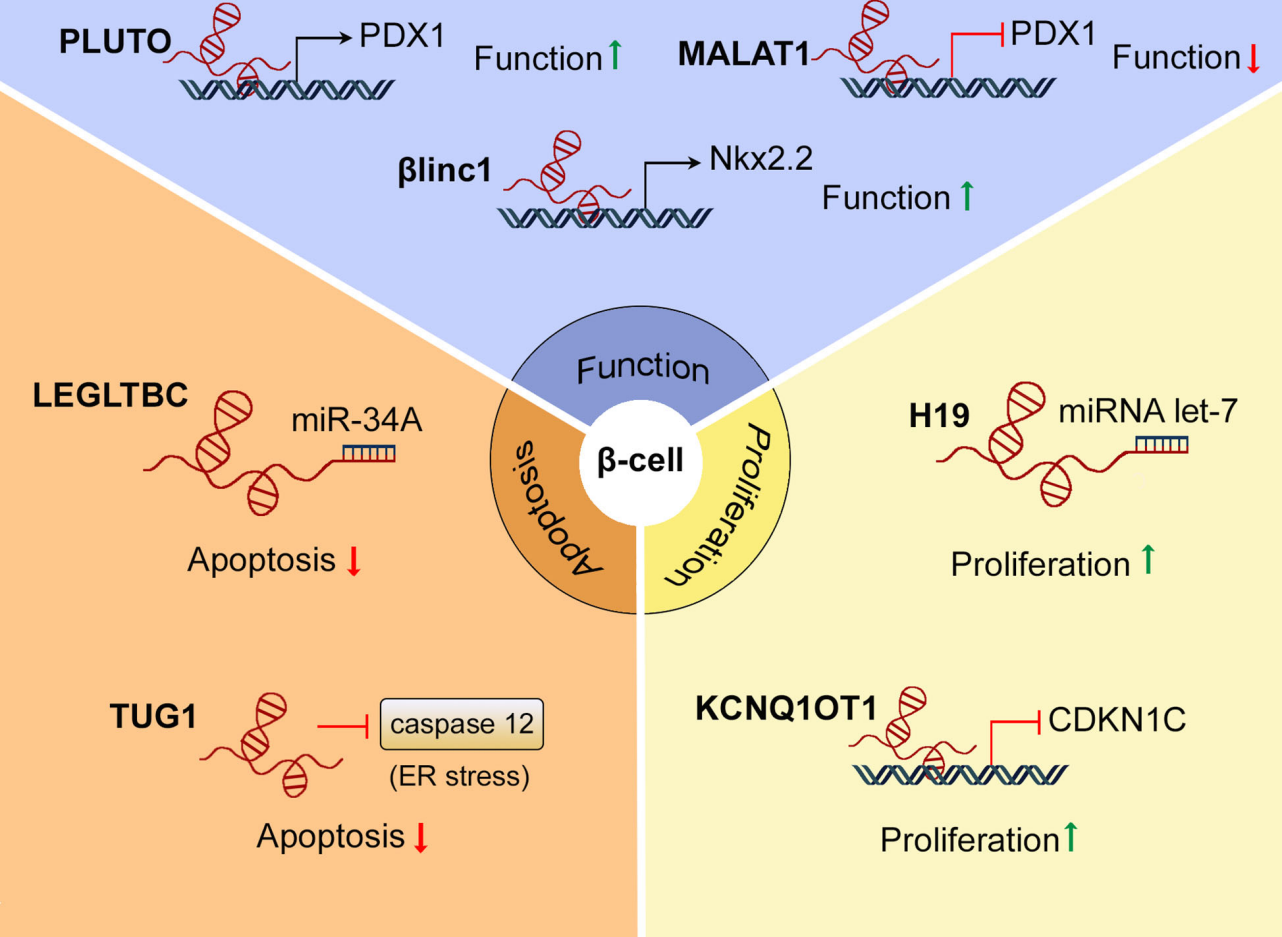
Roles of lncRNAs in β-cell mass and function. Several lncRNAs, including PLUTO, MALAT1, and βlinc1 have been found to regulate β-cell function by modulating the expression of β-cell signature genes. LncRNAs as LEGLTBC and TUG1 protect β-cells from apoptosis in response to different stimuli. H19 and KCNQ1OT1 are involved in β-cell mass expansion during development, promoting β-cell proliferation.

As stated earlier, a subgroup of patients with non-Mendelian forms of the disease develop T2D without showing significant insulin resistance suggesting that defects in β-cell mass and/or function are the principal disease drivers in these patients. In any case, *relative* insulin deficiency is a *sine qua non* for the emergence of the frank disease even in the presence of insulin resistance. In both instances, inadequate neonatal β-cell expansion may predispose to the appearance of diabetes later in life. Therefore, it is important to identify genetic factors driving β-cell proliferation during neonatal development. *H19* is a maternally imprinted intergenic lncRNA located at the *Igf2* locus that has recently been shown to drive β-cell mass expansion during development (73). Ding et al. (74) first described differential expression of *H19* in islets from the offspring of a mouse model for gestational diabetes (GD), which develop glucose intolerance and impaired glucose stimulated insulin secretion (GSIS) with age. Recently, *H19* was found to be required for normal β-cell mass expansion during the neonatal period through the activation of the Akt/PKB pathway. *H19* mediated β-cell proliferation was also dependent on the presence of Argonaute 2 (Ago2), an essential component of the RNA-induced silencing complex (RISC), suggesting that *H19* may act as a miRNA sponge in β-cells. Supporting this view, removal of binding sites in *H19* for the miRNA *let-7* reduced the proliferative capacities of the long non-coding RNA (**Figure 3**). Therefore, and although other mechanisms were not excluded, the authors proposed that *H19* may induce β-cell proliferation at least partially through inhibiting *let-7*, which in turns may activate the PI3K/Akt pathway, as it does in skeletal muscle. Importantly, although *H19* expression decreased considerably in adult islets compared to those from neonates, this lncRNA was re-expressed under conditions of high insulin demand. The latter findings suggest that *H19* may also be involved in β-cell mass compensation in rodents, although further studies are needed to assess the role of H19 in human β-cells (73).

GWAS have revealed that several single nucleotide polymorphisms (SNPs) in the *KCNQ1* region, containing the lncRNA *KCNQ1OT1*, are strongly associated with T2D risk (21, 75). In early development, *KCNQ1OT1* is expressed exclusively from the paternal allele and has been linked to regulate the imprinted expression of neighbouring target genes, including the Kv7.1 voltage-gated potassium channel subunit (*KCNQ1*) and cyclin-dependent kinase inhibitor 1C (*CDKN*1*C*), resulting in these genes being expressed exclusively from the maternal allele (**Figure 2**). However, the imprinting of *KCNQ1* is lost during adulthood, whereas *CDKN1C* expression remains monoallelic throughout life (76). Asahara et al. showed that truncation of the paternal allele of *Kcnq1ot1* in mice increased the expression of *Cdkn1c* in a tissue-specific manner, resulting in decreased β-cell mass at birth and at 24 weeks of age (**Figure 3**). Moreover, mice harbouring paternally-truncated *Kcnq1ot1* displayed glucose intolerance and defects in insulin secretion (77). It is therefore tempting to speculate that decreased expression of *KCNQ1OT1*, and subsequent upregulation of *CDKN1C*, mediates diabetes susceptibility at the *KCNQ1* locus.

In addition, several SNPs at the *CDKN2A/B* locus that encodes the lncRNA *ANRIL*, MTAP, and the cell cycle inhibitors *p14*, *p15*, and *p16*, have been linked to increased T2D risk. The homozygous risk variants of two of these SNPs, rs2383208 and rs10811661, were associated with high expression of *ANRIL* in samples from young donors (between 10 and 50 years old), while only protective variants showed an age-dependent increase. Moreover, the risk allele of rs564398, a SNP located within exon 2 of *ANRIL*, was associated with reduced β-cell proliferation rate. Therefore, these results suggest a potential role for *ANRIL* in the maintenance of human β-cell mass (78).

## LncRNAs in β-Cell Apoptosis

Numerous studies have shown that there is a significant reduction of β-cell mass in both T1D and T2D (79), although the extent of the “loss” of β-cell mass in T2D – usually reported as the difference in β-cell mass between populations of diabetic and control subjects; prospective measurements of β-cell mass in the same individual over time are not currently feasible (80) – is disputed, ranging from 25% to 50% (81). Although β-cell apoptosis is certainly a major aspect of the pathogenesis of T1D (32), there is a debate regarding the relative contributions of apoptosis, β-cell dedifferentiation and dysfunction, in the development of T2D (82).

Interestingly, several lncRNAs that have been shown to impact β-cell function also play an important role in β-cell apoptosis. Silencing the lncRNA Taurine-upregulated gene 1 (*TUG1*) was sufficient to induce β-cell apoptosis in MIN6 cells and pancreatic islets. This involved upregulated expression of the effector caspases 3 and 7 as well as caspase 12, which is associated with ER-stress-mediated apoptosis (**Figure 3**). In addition, *TUG1* knockdown also reduced *Ins1* and *Ins2* mRNA levels and impaired GSIS in MIN6 cells. Importantly, wild-type (wt) mice injected with an siRNA targeting *TUG1* showed reduced plasma insulin levels and increased fasting glycaemia (83). Similarly, the lncRNA Maternally expressed gene 3 (*Meg3*) has been shown to regulate insulin secretion as well as β-cell apoptosis (84). *Meg3* expression is down-regulated in pancreatic islets from T1D and T2D mouse models (NOD and hyperglycaemic *db/db* mice, respectively) (85) as well as in pancreatic islets from T2D donors (86). In addition, a SNP located within an intron of *Meg3* is strongly associated with T1D risk (87). *Meg3* down-regulation reduces the expression of ins2, *Pdx1* and *Mafa*, impairing GSIS both *in vitro* and *in vivo (* 85 *)*. Moreover, this manoeuvre upregulated the pro-apoptotic proteins Bax and caspase-3, increasing β-cell apoptosis. Furthermore, wild-type mice injected with an siRNA targeting *Meg3* display reduced β-cell mass (84). Nevertheless, despite the association studies linking *Meg3* expression with T1D and T2D in humans, there are no current studies assessing the impact of *Meg3* in human β-cells.

In contrast, Morlette et al. (35) identified two different lncRNAs that were differentially expressed in mice fed with a high fat diet (HFD), as well as in *db/db* mice, and which had an impact in β-cell apoptosis without affecting β-cell function. *βlinc2*, a lncRNA mapped in the mouse to chromosome 14, was up-regulated in pancreatic islets from T2D models and its expression was positively correlated with weight, glycaemia and hyperinsulinemia. The lncRNA *βlinc3* was downregulated in pancreatic islets from T2D mouse models and its expression was negatively correlated with weight and glycaemia. Remarkably, a human ortholog for *βlinc3* was also found to be downregulated in islets from T2D patients, while no orthologs were found for *βlinc2* in humans. Overexpression of *βlinc2*, or downregulation of *βlinc3*, had no effects on pro-insulin mRNA levels, total insulin content or GSIS. However, overexpression of *βlinc2* or knockdown of *βlinc3* increased β-cell apoptosis in Min6 cells (35).

A number of lncRNAs have also been shown to be involved in β-cell apoptosis mediated by different stress stimuli, such as glucolipotoxicity or inflammation. Kong et al. (88) identified ten different lncRNAs that were modulated in the rat β-cell line INS-1 after treatment with a combination of high glucose and palmitate. One of the lncRNAs identified, named *LEGLTBC* (low expression in glucolipotoxicity-treated beta cells), was shown to reduce oxidative stress and glucolipotoxicity-mediated β-cell apoptosis, acting as a sponge for miR-34a, which targets *SIRT1* (**Figure 3**). Correspondingly, silencing of *SIRT1* diminished the protective effects of *LEGLTBC* overexpression (89). In contrast, increased expression of the lncRNA PVT1 after streptozotozin (STZ) treatment, partially mediates the increase in oxidative stress and apoptosis induced by the drug, while knocking down PVT1 reduces STZ-mediated β-cell apoptosis (90). In another study (32), the expression of several lncRNAs was found to be up-regulated in MIN6 cells and mouse islets after 24 h of cytokine treatment. Importantly, the expression of three of these lncRNAs was also modulated during the progression of insulitis in non-obese diabetic (NOD) mice, suggesting a role for these lncRNAs in inflammatory processes. Overexpression of these lncRNAs in MIN6 cells did not affect insulin content or secretion, but sensitized β-cells to apoptosis. Interestingly, the overexpression of one of the lncRNAs [referred as lncRNA-1 in the study (32)] promoted the translocation of NF-κB to the nucleus to a similar extent as treatment with interleukin 1 β (IL-1β) and interferon-γ (IFN-γ), indicating a role for this lncRNA in cytokine-mediated apoptosis (32). More recently, a lncRNA associated with T1D, *Lnc13*, was shown to promote *STAT1* mRNA stability (91), the latter considered a master modulator of inflammation-mediated β-cell apoptosis (92). Several studies have identified a number of SNPs located on chromosome 12q13, surrounding the *ERBB3* gene and its antisense lncRNA NONHSAG011351, that are associated with T1D risk. NONHSAG011351 may regulate *ERBB3* expression, which is downregulated in human islets after exposure to pro-inflammatory cytokines. Furthermore, knocking down *Erbb3* in INS1-cells reduced cytokine-induced apoptosis, indicating a role for ERBB3 and possibly NONHSAG011351 in modulating β-cell survival in response to inflammation (93).

## LncRNAs in β-Cell Identity and Function

For a number of years, it was believed that β-cell apoptosis played a major role in the pathogenesis of T2D (6, 66). However, later studies (82) indicated that the apoptosis level detected in pancreata from T2D donors did not correlate with the decline in β-cell mass and function observed. During the last decade, loss of β-cell identity has increasingly been recognized as a major contributor to compromised insulin secretion in T2D (10, 82). Moreover, several studies have also reported that a decrease in β-cell function precedes β-cell mass decline in T1D (94, 95). Indeed, defects in β-cell secretory responses to glucose have been reported in T1D patients five years before the diagnosis of the disease (96). As noted in the Introduction, maintenance of fully differentiated and functional β-cells requires the expression of key β-cell transcription factors, including *Pdx1* (97), *MafA (* 98), *Nkx6.1*, *Nkx2.2*, and *Pax6* (99), alongside with the inhibition of the so-called “disallowed” or “forbidden” genes, such as *Ldha* and *Hsd11b1* (12, 13). Changes in the expression of any or all of the above may therefore contribute to impaired β-cell identity in T2D (13, 15). Importantly, several of these critical genes appear to be the target of lncRNA action.

Many of the lncRNAs identified in pancreatic islets are mapped to the proximity of transcription factors required for β-cell function and maturation. A number of these lncRNAs have been shown to regulate these transcription factors in *cis* (36). The lncRNA *PLUTO*, transcribed antisense to *Pdx1*, modulates the expression of the latter transcription factor by promoting the interaction of an enhancer region within its promoter (**Figure 2**). Consistent with a downregulation of *Pdx1*, silencing of PLUTO in human-derived EndoC-βH3 cells impaired GSIS (**Figure 3**) (36). Another islet specific lncRNA, known as *βlinc1* in mouse and *HI-LNC15* in humans, has been shown to regulate the expression of *Nkx2.2* in *cis*. The lncRNA *βlinc1/HI-LNC15* is mapped in an intergenic region between *Nkx2-2* and *Pax1* on chromosome 2 in mice and chromosome 20 in humans. Although the specific mechanism(s) of action of *βlinc1/HI-LNC15* remains to be elucidated, silencing of this lncRNA in MIN6 and EndoC-βH1 cells resulted in the down-regulation of *Nkx2-2* together with a large number of *Nkx2-2* target genes (**Figure 3**). Moreover, mice null for *βlinc1* are glucose intolerant and display defects in pancreatic endocrine cell lineage specification, showing a similar phenotype to *Nkx2-2* knockout (KO) mice (100). However, a functional role for HI-LNC15 in human β-cells still remains to be confirmed.

Two different lncRNAs, *Paupar* and *Pax6os1/PAX6-AS1*, are transcribed antisense from the *Pax6* locus (101, 102). PAX6 is another transcription factor required for β-cell differentiation in the embryo and β-cell function during adulthood (103, 104). *Paupar* is expressed predominantly in α-cells within the islets, where it acts in *cis* to regulate the alternative splicing of *Pax6*. Paupar-silencing decreases the expression of the *Pax6* isoform *Pax6 5a*, resulting in decreased expression of several α-cell signature genes and defective glucagon secretion (101). In contrast, knockdown of *Pax6os1/PAX6-AS1*, which is more abundant in β than α cells, up-regulates insulin (*Ins1*) expression in MIN6 and human EndoC-βH1 cells and enhances GSIS in human islets (102). Interestingly, *Paupar* is down-regulated in islets from *db/db* mice (101), while *Pax6os1/PAX6-AS1* expression is increased in islets from mice fed a HFD and in human T2D donors *versus* non-diabetic controls (102).

A number of lncRNAs have also been shown to regulate the expression of β-cell signature transcription factors in *trans* (53, 105). As indicated above, *MALAT1* downregulates *Pdx1* expression by inhibiting H3 histone acetylation in its promoter region (**Figure 3**). Ding et al. (53) showed that IL-1β treatment down-regulated *Pdx1* and impaired GSIS in MIN6 cells, while knocking down *MALAT1* restored both *Pdx1* levels and insulin secretion in response to glucose. Interestingly, *MALAT1* expression was induced by IL-1β treatment and during the progression of hyperglycaemia in the non-obese diabetic (NOD) T1D mouse model, indicating that this lncRNA might play a role in the decline of β-cell function during the progression of T1D. In the context of T2D, however, silencing *MALAT1* has been suggested to impair β-cell function, reducing GSIS and increasing β-cell apoptosis. Xiong et al (40). reported a decrease in *MALAT1* expression in *db/db* mice as well as MIN6 cells treated with palmitate. In this study, *MALAT1* was found to inhibit Polypyrimidine tract binding protein (Ptbp) protein degradation in the nucleus, which, according to the authors, mediated the increased insulin expression and reduced β-cell apoptosis observed after exendin-4 treatment. In contrast, Sun et al. (54) reported that increased levels of *MALAT1* contributed to β-cell dysfunction caused by cigarette smoke. These authors found that *MALAT1* acts as a sponge for mir-17, which inhibits *TXNIP* expression, the latter serving as an important regulator of β-cell survival and function (106). Thus, increased levels of MALAT1 were found to decrease mir-17 expression, increase TXNIP mRNA levels and decrease the expression of MAFA. Silencing *MALAT1* in MIN6 cells down-regulated *TXNIP*, recovered mir-17 and MAFA expression, increased insulin content and enhanced GSIS (54). Nevertheless, the role of MALAT1 in β-cell function under different physiological circumstances and in human β-cells is not fully understood and should be clarified before proposing this lncRNA as a possible target to treat diabetes.

*NKX6.1* is another transcription factor required for maintaining functional and fully mature β-cells and *Nkx6.1/NKX6.1* expression is decreased during the development of T2D in both rodents and humans (107). Recently, two lncRNAs, *810019D21Rik* (also referred as *ROIT*) and Gm10451, were shown to regulate *Nkx6.1* expression in *trans* by modulating different epigenetic marks. ROIT, which is transcribed antisense to *Espr2* from a locus syntenic block on human chromosome 16 and mouse chromosome 8, promotes the degradation of DNA methyltransferase 3A (DNMT3A), thus affecting Nkx6.1 methylation. Knocking down ROIT, decreased insulin expression and impaired GSIS in MIN6 cells, while ROIT overexpression upregulated insulin and enhanced GSIS *in vitro* (105). In addition, inhibition of ROIT in wt mice, impaired insulin synthesis and glucose tolerance. Remarkably, *ROIT* expression was down-regulated in islets from multiple mouse models of T2D (HFD-fed, *db/db* and *ob/ob* mice) as well as in the serum of patients with T2D (105). These data provide compelling evidence of a role for ROIT in the development of the disease. The lncRNA Gm10451 seems to regulate *Nkx6.1* expression during development by targeting miR-338-3p as a competitive endogenous RNA (cerna). This microRNA regulates the expression of the histone H3K4 methyltransferase complex PTIP (Pax Transcription activation domain Interacting Protein), which is required for appropriate expression of Nkx6.1, along with other mature β-cell markers such as Insulin and MafA (108).

Reduced levels of the lncRNA GAS5 (growth arrest-specific transcript 5) in human serum have been correlated with T2D (109). Moreover, GAS5 expression has been found to be downregulated in islets from *db/db* mice (110). Decreased expression of this lncRNA in MIN6 cells reduces insulin synthesis and secretion, pointing to a role for GAS5 in β-cell dysfunction during T2D (110). Interestingly, glucocorticoid treatment impairs insulin secretion and reduces the expression of GAS5 in EndoC-βH1 cells cultured at high glucose concentrations. Introducing the active segment of GAS5, called the hormone response element mimic (HREM), rescued GSIS in glucocorticoid-treated cells, suggesting that GAS5 could modulate the glucocorticoid-glucocorticoid receptor (GC-GR) pathway (33). In Hela cells, GAS5 has been shown to act as a decoy for the glucocorticoid receptor that acts as a ligand-dependent transcription factor since it binds to the DNA binding domain of the protein (**Figure 2**) (111). As opposed to the previous study, however, the latter authors found that GAS5 expression was up-regulated in the islets of T2D donors and in a rat model of T2D. Since GAS5 down-regulation robustly impaired β-cell function, the authors hypothesized that GAS5 induction might be part of a β-cell compensatory mechanism to hyperglycaemia (33). Similarly, the lncRNA p3134 may also play a role in β-cell compensation in diabetes. Indeed, p3134 expression is increased in the blood of T2D patients, while overexpressing this lncRNA has been shown to enhance GSIS in MIN6 cells and restore β-cell function *db/db* mice (112). In contrast, knocking down the lncRNA linc-p21, whose levels are also increased in the serum of T2D versus normoglycemic patients, enhances GSIS and β-cell proliferation, while overexpressing it has opposite effects (113).

## Conclusions

We argue here that understanding the functions of non-coding RNAs in β-cell biology may provide crucial insights into the pathogenesis of T1D and T2D. Over the past decade, numerous studies have indicated that lncRNAs play important roles in the maintenance of β-cell mass and function. Importantly, several long non-coding RNAs that are differentially expressed during diabetes progression have also been shown to regulate β-cell proliferation, apoptosis, or to modulate the expression of genes required for a fully functional and mature β-cell phenotype (36, 84, 102). A summary of these roles is provided in **Figure 3**.

The highly cell- or tissue-specific expression profile of lncRNAs make them exceptional therapeutic targets, as they could – in theory – be specifically targeted in a given cell-type, limiting off-target effects (114). Furthermore, sequence-based nucleic acid therapeutics (such as antisense oligonucleotides) are evolving rapidly, and several oligonucleotide-based drugs have already been approved by the U.S. Food and Drug Administration (FDA) (115, 116). Clinical trials of novel treatments targeting lncRNAs are already being conducted in the context of other diseases, such as cancer. For instance, BC-819, which is a DNA plasmid that drives the expression of the diphtheria toxin gene under the regulation of the H19 promoter, has been tested in several trials as a treatment for cancers overexpressing H19, including pancreatic cancer (117). However, a better understanding of the roles of specific lncRNAs in β-cell biology, and their mechanisms of action, is needed before they can be considered as therapeutic targets. This point is illustrated by the conflicting results obtained for *MALAT1* downregulation in β-cells under different pathophysiological conditions (40, 53, 54). In addition, some lncRNAs may play redundant functions in β-cells. For example, the lncRNA A830019P07Rik is differentially expressed in pancreatic islets during different stages of development and its expression is reduced in pancreatic islets from both *db/db* and *ob/ob* mice. However, mice null for this lncRNA do not display any changes in glucose tolerance or insulin secretion (34). Likewise, knocking down the β-cell specific lncRNA G3R1 does not affect glucose metabolism or islet architecture in mice (118). Furthermore, many functional studies of lncRNAs have only been performed in rodent cell lines up to now, although a few have been undertaken in human-derived β-cells, such as EndoC-βH1 (e.g., PLUTO and PAX6-AS1). It will be important, therefore, to validate these findings in human systems.

In conclusion, although several lncRNAs have been shown to play important roles in β-cell function and survival, further detailed investigations of their mechanism of action are needed before they can be considered *bona fide* therapeutic targets to treat T1D or T2D.

## Author Contributions

LL and GR jointly conceived and wrote the manuscript. LL provided the original draft of the article and prepared the figures. All authors contributed to the article and approved the submitted version.

## Funding

GAR was supported by a Wellcome Trust Investigator Award (212625/Z/18/Z), MRC Programme grants (MR/R022259/1, MR/J0003042/1, MR/L020149/1) and by Diabetes UK (BDA/11/0004210, BDA/15/0005275, BDA 16/0005485) project grants. This project has received funding from the European Union’s Horizon 2020 research and innovation programme *via* the Innovative Medicines Initiative 2 Joint Undertaking under grant agreement No 115881 (RHAPSODY). This Joint Undertaking receives support from the European Union’s Horizon 2020 research and innovation programme and EFPIA.

## Conflict of Interest

GR has received research grant funding from Sun Pharmaceuticals Inc and Les Laboratoires Servier, and is a consultant for Sun Pharmaceuticals. These funders had no involvement with the present study.

The remaining author declares that the research was conducted in the absence of any commercial or financial relationships that could be construed as a potential conflict of interest.

## Abbreviations

FDA, U.S. Food and Drug Administration; GD, Gestational diabetes; GSIS, Glucose stimulated insulin secretion; GWAS, Genome wide-association studies; HFD, High fat diet; KO, knockout; LncRNAs, Long non-coding RNAs; miRNA, micro-RNA; MODY, maturity onset diabetes of the young; NcRNAS, non-coding RNAs; NOD, non-obese diabetic; Nts, nucleotides; SNP, single nucleotide polymorphism; STZ, streptozotocin; T1D, Type 1 diabetes; T2D, Type 2 diabetes; WT, wild-type.
